# Dentigerous Cyst Associated with a Mesiodens: A Case Report

**DOI:** 10.5681/joddd.2011.016

**Published:** 2011-06-14

**Authors:** Sepideh Vosough Hosseini, Monir Moradzadeh, Mehrdad Lotfi, Amir Ala Aghbali, Shirin Fattahi

**Affiliations:** ^1^ Associate Professor, Department of Oral and Maxillofacial Pathology, Faculty of Dentistry, Tabriz University of Medical Sciences, Tabriz, Iran; ^2^ Assistant Professor, Department of Oral and Maxillofacial Pathology, Faculty of Dentistry, Tabriz University of Medical Sciences, Tabriz, Iran; ^3^ Associate Professor, Department of Endodontics, Faculty of Dentistry, Tabriz University of Medical Sciences, Tabriz, Iran; ^4^ Postgraduate Student, Department of Oral and Maxillofacial Pathology, Faculty of Dentistry, Tabriz University of Medical Sciences, Tabriz, Iran

**Keywords:** Cholesterol cleft, dentigerous cyst, mesiodens, supernumerary tooth

## Abstract

Dentigerous cysts are the second most common odontogenic cysts after radicular cysts and are most commonly seen in association with third molars and maxillary canines. Only 5% of dentigerous cysts involve supernumerary teeth, of which mesiodens is the most frequent type. This paper presents a case of dentigerous cyst associated with a mesiodens that caused a painless swelling in the upper lip of an 18-year-old female. The patient was treated surgically by enucleation of total cyst and surgical extraction of mesiodens under local anesthesia.

## Introduction

Dentigerous or follicular cysts are the second most common type of odontogenic cysts, and the most common developmental cysts of the jaws.^1^The dentigerous cyst is defined as a cyst that originates by the separation of the follicle from around the crown of an unerupted tooth.^2^Dental follicle associated with unerupted or impacted teeth shows fibrous connective tissue with remnants of reduced enamel epithelium. Dentigerous cysts are caused by expansion of dental follicles resulting from accumulation of fluid between tooth crowns and epithelial components.^3^

This cyst most frequently occurs in patients between 10 and 30 years of ages and there is a greater incidence in males with a 1.6:1 ratio.^1,2^ The cysts most often involve impacted mandibular third molars, followed by maxillary canines, mandibular premolars, and occasionally supernumerary teeth or odontomas.^2,4^

The clinical examination reveals a missing tooth or teeth and occasionally a hard swelling, sometimes resulting in facial asymmetry and possible pathologic fracture. Dentigerous cysts are tentatively diagnosed on routine dental radiographs.^5^ Radiographically, the cyst appears as a unilocular radiolucent shadow with a well-defined sclerotic border associated with the crown of an unerupted tooth, but an infected cyst will show ill-defined borders.^6^

Only 5% of dentigerous cysts are associated with supernumerary teeth. The usual age of clinical presentation of dentigerous cyst due to supernumerary tooth is in the first four decades of life.^7^

Supernumerary teeth are most common in the maxilla with a strong predilection for the anterior region, in which case the supernumerary tooth is termed a mesiodens.^1,2^

This paper presents a case of dentigerous cyst associated with a mesiodens in an 18-year- old female.

## Case Report

An 18-year-old female patient presented to the Department of Oral and Maxillofacial Surgery, Faculty of Dentistry at Tabriz University of Medical Sciences, Tabriz, Iran, with the chief compliant of a painless swelling in the upper jaw, which had been gradually increasing in size for the one year. At the time of her presenting, the patient had no systemic disease.

The intraoral clinical examination detected a firm labial and palatal swelling in the maxillary anterior region, on the both sides of the midline.

**Figure 1:**
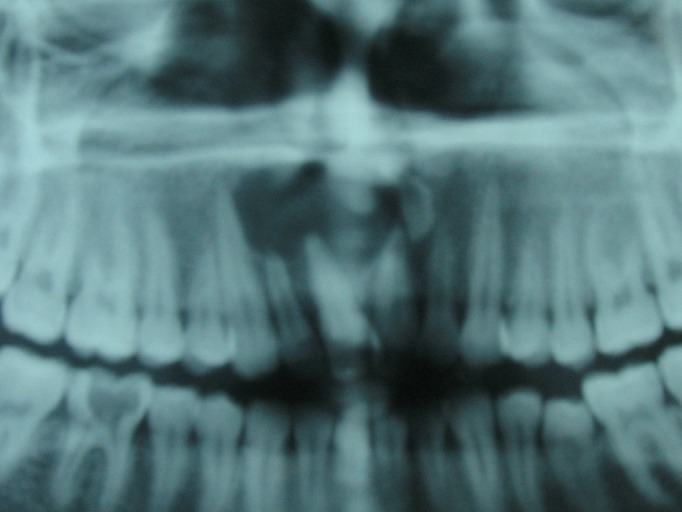
Panoramic radiograph shows a large unilocular radiolucency in the anterior maxilla and the mesiodens in the left aspect of the lesion.

The panoramic radiograph showed a large, well-defined radiolucent lesion with sclerotic borders. The lesion extended from the right canine to the left lateral incisor region. A supernumerary tooth was visible in the left aspect of the cyst, resulting in resorption of cortical bone at this region. Divergence of the roots of the central incisors was also noted on the radiograph. The supernumerary tooth had a cone-shaped crown and a shortened root (Figure 1).

The lesion was totally enucleated together with the supernumerary tooth under local anesthesia, and specimens were sent to the Department of Oral and Maxillofacial Pathology, with the differential diagnoses of periapical cyst, odontogenic keratocyst, and adenomatoid odontogenic tumor. The submitted specimens consisted of two fragments of soft tissue, the largest measuring approximately 21 mm ? 13 mm ? 3 mm.

**Figure 2:**
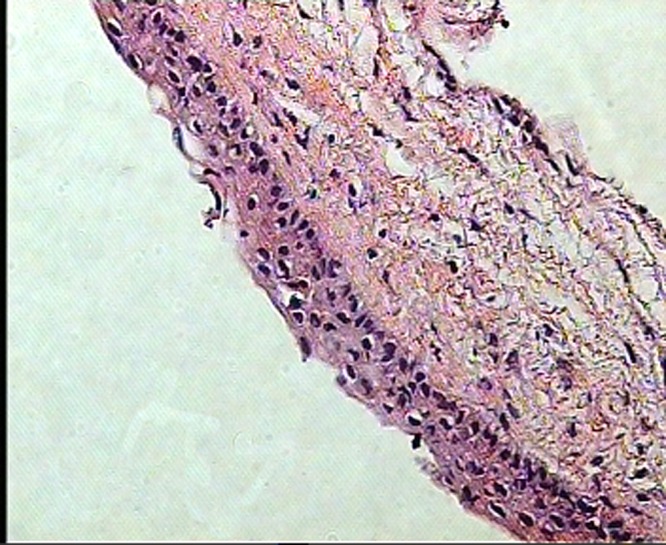
Photomigrograph showing cystic wall with non-keratinizing squamous epithelium (haematoxylin-eosin staining, ?20)

Histological sections of both specimens revealed cyst walls composed of loosely arranged fibrovascular connective tissue, lined by 2-4 layers of flattened non-keratinizing stratified squamous epithelium (Figure 2). The epithelium and the connective tissue interface was flat. Numerous cholesterol clefts and few chronic inflammatory cells infiltration were noted. No evidence of malignant changes was noted.

The clinical, radiographic and histopathological features led to the final diagnosis of dentigerous cyst associated with a mesiodens.

The patient was followed up for six months. The post-operative period was uneventful.

## Discussion

Swelling of the upper lip may result from different conditions including infections, allergic diseases, neoplasms (especially of salivary origin), granulomatous diseases, and different types of cysts.^8^

Dentigerous cyst is a common oral lesion caused by fluid accumulation between the reduced enamel epithelium and the crown of an unerupted tooth.^2^ Most reports have shown a peak incidence of dentigerous cysts in the second and third decades of life, with a slightly higher predilection for males .This gender preference might be related to a smaller jaw size and a greater tendency for prophylactic extraction of third molars in females .^9^

A panoramic radiograph of a patient with dentigerous cyst reveals a unilocular radiolucency enclosing the crown of an unerupted tooth. The radiolucency usually arises in the cemento-enamel junction of the tooth.^9^If a follicular space on the radiograph is more than 5 mm, an odontogenic cyst can be suspected. Differential diagnoses of such radiolucency include radicular cyst, odontogenic keratocyst, and odontogenic tumors such as ameloblastoma, Pindborg tumor, odontoma, odontogenic fibroma, and cementomas.^10^

Although dentigerous cyst is a common developmental cyst, its association with supernumerary teeth is rare and estimated to constitute 5-6% of all dentigerous cysts.^4^ First named by Bolk in 1917, mesiodens is the most frequent type of supernumerary teeth and situated in the maxillary anterior incisors region. It is a rare entity with a prevalence of 0.15-1.9% in general population and a slight male predilection.^2,8,11^ Mesiodens may be single or multiple, erupted or impacted and is rarely seen associated with a dentigerous cyst.^12^ Scolozzi et al^8^ reported an unusual case of a large dentigerous cyst associated with an impacted mesiodens, resulting in a slow-growing swelling in the upper lip. Khan et al^13^ also described an upper lip swelling caused by a large dentigerous cyst associated with mesiodens. Dinkar et al^14^described an unusually early presentation of multiple mesiodens with associated dentigerous cyst. Large dentigerous cysts arising from unerupted mesiodens should be kept in mind in the differential diagnosis of upper lip swellings, particularly if associated with dental anomalies of the maxillary incisors such as malposition and diastema.

Sharma et al^15^ reported the case of a dentigerous cyst associated with an impacted inverted mesiodens that developed secondary to trauma to its predecessor, a non-vital permanent maxillary central incisor. As a consequence of trauma, the central incisor?s root development was prematurely arrested and the open apex lied close to the follicle of the underlying inverted mesiodens.

The presence of mesiodens causes complications such as delayed eruption of permanent teeth, rotations, retention, root resorption, pulp necrosis, and diastema as well as formation of dentigerous and primordial cysts.^16^

Therefore, to prevent the development of a dentigerous cyst and to avoid unwanted effects on neighboring teeth, early diagnosis and treatment of mesiodens are crucial.
